# *FvWRKY50* is an important gene that regulates both vegetative growth and reproductive growth in strawberry

**DOI:** 10.1093/hr/uhad115

**Published:** 2023-05-31

**Authors:** Yating Chen, Liping Liu, Qianqian Feng, Chuang Liu, Yujuan Bao, Nan Zhang, Ronghui Sun, Zhaonan Yin, Chuanfei Zhong, Yuanhua Wang, Qian Li, Bingbing Li

**Affiliations:** Department of Pomology, College of Horticulture, China Agricultural University, Beijing, 10093, China; Department of Pomology, College of Horticulture, China Agricultural University, Beijing, 10093, China; Department of Pomology, College of Horticulture, China Agricultural University, Beijing, 10093, China; Department of Pomology, College of Horticulture, China Agricultural University, Beijing, 10093, China; Department of Pomology, College of Horticulture, China Agricultural University, Beijing, 10093, China; Department of Pomology, College of Horticulture, China Agricultural University, Beijing, 10093, China; Department of Pomology, College of Horticulture, China Agricultural University, Beijing, 10093, China; Department of Pomology, College of Horticulture, China Agricultural University, Beijing, 10093, China; Beijing Engineering Research Center for Strawberry, Institute of Forestry and Pomology, Beijing Academy of Agriculture and Forestry Sciences, Beijing, 100093, China; Department of Agronomy and Horticulture, Jiangsu Vocational College of Agriculture and Forestry, Jiangsu, 212400, China; Engineering and Technical Center for Modern Horticulture, Jiangsu, 212400, China; Department of Pomology, College of Horticulture, China Agricultural University, Beijing, 10093, China; Department of Pomology, College of Horticulture, China Agricultural University, Beijing, 10093, China

## Abstract

The WRKY transcription factors play important roles in plant growth and resistance, but only a few members have been identified in strawberry. Here we identified a WRKY transcription factor, FvWRKY50, in diploid strawberry which played essential roles in strawberry vegetative growth, and reproductive growth. Knocking out *FvWRKY50* by genome editing accelerated flowering time and leaf senescence but delayed anthocyanin accumulation in fruit. Further analysis showed that FvWRKY50 acted as a transcriptional repressor to negatively regulate the expression of flowering- and leaf senescence-related genes, including *FvFT2*, *FvCO*, *FvFT3*, and *FvSAUR36*. Notably, FvWRKY50 directly upregulated the expression of *FvCHI* and *FvDFR* by binding their promoter under normal conditions, but at low temperature FvWRKY50 was phosphorylated by FvMAPK3 and then induced protein degradation by ubiquitination, delaying anthocyanin accumulation. In addition, the homozygous mutant of *FvWRKY50* was smaller while the biallelic mutant showed normal size. These new findings provide important clues for us to further reveal the regulatory mechanisms of strawberry growth and fruit ripening

## Introduction

Strawberry (*Fragaria* × *ananassa* Duch.) is one of the most important fruit crops worldwide because of its unique flavor and high nutritional value. Strawberry fruit is non-climacteric because its respiration rate and ethylene production have not dramatically changed during fruit development and ripening [5, 38]. Fruit ripening is a very complex process during which various biochemical and physiological changes happen in various traits, including fruit color, texture, aroma, and other quality aspects [17, 21, 36]. During the complex fruit ripening progress transcriptional regulation is indispensable because expressions of the ripening-related genes are modulated by various transcription factors [23, 35,56].

Previous studies have reported some transcription factors that could regulate strawberry fruit development and ripening, including RIF (an NAC transcription factor), TCP9 (a TCP transcription factor), WRKY48 and WRKY71 (WRKY transcription factors), MYB10, MYB44.2 and MYB79 (MYB transcription factors), RAV (an AP2/ERF transcription factor), and MADS9 [1, 3, 4, 22, 27, 39, 43, 48, 51, 52]. Among the different kinds of transcription factors, plant-specific WRKY transcription factors play an important role in the process of strawberry fruit ripening. However, to date only a few members of the WRKY transcription factors have been reported, and most of them are not well known in strawberry.

There are always one or two conserved WRKY domains in WRKY proteins that can bind the W-box of target gene promoter sequences to regulate their expression in various physiological processes [2, 32, 34]. It is well known that WRKY transcription factors can modulate defensive responses to biotic or abiotic stresses [13, 42]. WRKY transcription factor family genes have been analyzed in the diploid woodland strawberry and their expression patterns in various stress responses or the fruit development process have been determined [46, 53]. Among them, *FvWRKY42* was reported to regulate various stress responses, and overexpression of *FvWRKY42* in *Arabidopsis thaliana* could affect powdery mildew infection resistance and salt stress response in plants [45]. *FaWRKY25* and *FvWRKY50* (the homolog gene of *AtWRKY50*) were reported to regulate resistance to *Botrytis cinerea* in strawberry fruits [14, 25]. WRKYs were also found to function in the plant development and fruit ripening processes by regulating related target genes. For example, overexpression *FvWRKY71* in woodland strawberry could promote flowering by directly activating *FvFUL*, *FvAGL42*, *FvSEP1*, *FvLFY*, and *FvFPF1* [22], while transient overexpression of *FaWRKY71* in octoploid strawberry could affect strawberry fruit ripening [49]. FvWRKY48 could regulate strawberry fruit softening through directly binding to the pectate lyase *FvPLA* promoter, resulting in pectin degradation [51]. However, besides FvWRKY48 and FaWRKY71, knowledge of the roles of other WRKY transcription factors in strawberry fruit ripening is still limited.

Numerous external environmental factors and internal regulators were regarded as signals to induce transcription factor function in the transcriptional regulation network [19]. MITOGEN-ACTIVATED PROTEIN KINASE (MAPK) cascades were reported to govern diverse biological functions by activating downstream response factors like kinases and transcription factors [11, 18, 41] . A typical MAPK cascade is composed of three consecutive kinases, MAPK kinase kinases (MKKKs), MKKs, and MAPKs. In diploid woodland strawberry 12 *MAPK* genes have been identified [44, 54]. Strawberry fruit is highly sensitive to temperature and low temperature results in poor color of strawberry fruits. Our previous work indicated that FvMAPK3 could be activated by low temperature. On the one hand, FvMAPK3 phosphorylated and reduced the activity of FvMYB10. On the other hand, FvMAPK3 phosphorylated and degraded the FvCHS1 protein, both of which repressed the anthocyanin accumulation of strawberry fruits during the ripening process [26]. Besides FvMAPK3, a previous study also demonstrated that FaSnRK2.6 acted as a negative regulator to regulate strawberry fruit ripening [10]. However, whether FvMAPKs participate in the WRKY transcriptional regulation pathway in the strawberry fruit ripening process has not been explored.

Previously, *FvWRKY50* (the homolog gene of *AtWRKY75*) was reported to be induced by various stresses and hormone signals; however, its biological function remained unknown [46]. In this study we analyzed the function of *FvWRKY50* by generating gene-edited mutants in the diploid woodland strawberry ‘di Bosco’. The results demonstrated that *FvWRKY50* played essential roles in plant growth, leaf senescence, flowering and fruit ripening in strawberry. In addition, FvWRKY50 could be phosphorylated and degraded by FvMAPK3 to regulate anthocyanin accumulation in strawberry fruit at low temperature.

## Results

### Identification of *FvWRKY50* characteristics in diploid strawberry plants

Previous study has showed that *FvWRKY50* belongs to subgroup IIc of the FvWRKY family and responds dramatically to various biotic and abiotic stresses in the diploid strawberry ‘Heilongjiang-3’ [46]. To fully explore the function of *FvWRKY50* in strawberry, we further analyzed the bioinformatics and expression characteristic of *FvWRKY50* using the diploid strawberry ‘di Bosco’.

**Figure 1:**
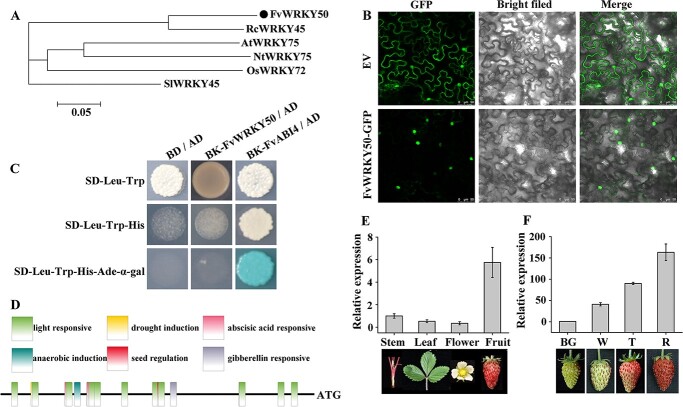
Bioinformatics and expression characteristic of *FvWRKY50*. (A) Phylogenetic analysis of WRKY50 in strawberry and other species. Fv, *F. vesca*; Rc, *R. chinensis*; At, *A. thaliana*; Nt, *N. tabacum*; Os, *Oryza sativa*; Sl, *Solanum lycopersicum*. (B) Subcellular localization of FvWRKY50. pSuper:GFP (top) or pSuper:*FvWRKY50*-GFP (bottom) was transformed into tobacco and the fluorescence signal was observed. (C) Transcriptional activation activity analysis of FvWRKY50 in yeast. (D) Motif analysis of the *FvWRKY50* promoter. (E) *FvWRKY50* gene expression in stem, leaf, flower, and fruit of diploid strawberry. qRT–PCR was used to determine the expression levels of *FvWRKY50*. (F) *FvWRKY50* gene expression pattern in the strawberry fruit developmental process. Diploid strawberry fruits were harvested at big green (BG), white (W), turning (T), and red ripening (R) stages and qRT–PCR was used to analyze the expression levels of *FvWRKY50.*

To examine the evolutionary relationship between FvWRKY50 and its orthologs from other species, a phylogenetic tree was constructed by neighbor-joining analysis, which indicated that FvWRKY50 had a close genetic relationship to the ortholog RcWRKY45 from *Rosa chinensis* and AtWRKY75 from *A. thaliana* (Fig. 1A).

To identify the subcellular localization of FvWRKY50 protein, FvWRKY50-GFP recombinant protein and GFP control protein were expressed in leaves of *Nicotiana benthamiana*. The fluorescence signal indicated that FvWRKY50 protein localized only in the nucleus and it was consistent with FvWRKY50 as a transcription factor (Fig. 1B). We further detected the activation activity of FvWRKY50 by transforming pGBKT7-FvWRKY50 into yeast cells and culturing on SD/−Leu−Trp, SD/−Leu−Trp−His, and SD/−Leu−Trp−His−Ade medium. pGBKT7-FvABI4 was used as the positive control. As shown in Fig. 1C, pGBKT7-FvWRKY50 could not grow while pGBKT7-FvABI4 grew well on different mediums, indicating that FvWRKY50 has no independent transcriptional activation activity. We then analyzed the sequence of the *FvWRKY50* promoter; several *cis*-acting regulatory elements involved in light responsiveness, abscisic acid responsiveness, anaerobic induction, gibberellin response, and seed regulation were identified (Fig. 1D). All these results suggested that FvWRKY50 may be involved in the regulation of various physiological processes and may need to recruit other transcription factors to perform transcriptional activation functions.

We further identified the expression pattern of *FvWRKY50* in different tissues and during different developmental stages of fruit in diploid strawberry. Real-time quantitative PCR (qRT–PCR) was used to determine the expression level of *FvWRKY50* in stem, leaf, flower, and fruit (Fig. 1E). The expression level of *FvWRKY50* increased gradually from big green fruit and showed the highest level in red fruit, indicating that *FvWRKY50* may be related to the regulation of strawberry fruit ripening (Fig. 1F).

### *FvWRKY50* regulated strawberry plant growth and auxin biosynthesis

**Figure 2:**
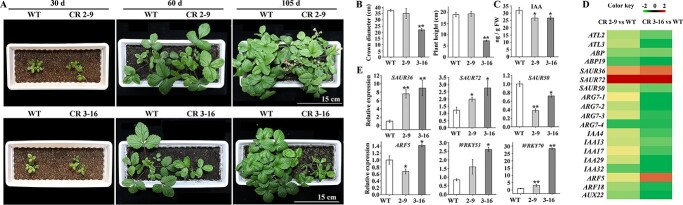
*FvWRKY50* regulated auxin biosynthesis and signaling transduction. (A) Phenotypes of WT, *FvWRKY50* CR 2-9, and *FvWRKY50* CR 3-16 in growth chambers. Seedlings of 30, 60, and 105 days were observed and photographed. (B) Crown diameter and plant height of WT, *FvWRKY50* CR 2-9, and *FvWRKY50* CR 3-16 plants at the same stage. (C) Auxin content of WT, *FvWRKY50* CR 2-9, and *FvWRKY50* CR 3-16 leaves. (D) Expression profiles of genes in auxin biosynthesis and signaling transduction pathways regulated by *FvWRKY50*. The heat map is based on transcriptome sequencing data of WT, *FvWRKY50* CR 2-9, and *FvWRKY50* CR 3-16 leaves. (E) Expression levels of partial DEGs confirmed by qRT–PCR.

To fully analyze the biological functions of *FvWRKY50* in strawberry, we used CRISPR/Cas9 to edit *FvWRKY50* in ‘di Bosco’ strawberry (Supplementary Data Fig. S1A). As shown in Supplementary Data Fig. S1B, two genome-edited lines were obtained. The biallelic mutant FvWRKY50 CR 2-9 has a deletion of 5-bp/131-bp in one allele and a deletion of 114-bp in the other allele, respectively. The homozygous mutant *FvWRKY50* CR 3-16 has a deletion of 114-bp in both alleles (Supplementary Data Fig. S1B). Surprisingly, we noticed that *FvWRKY50* CR 3-16 grew slowly and showed a distinct dwarf phenotype while *FvWRKY50* CR 2-9 showed no significant difference compared with wild-type (WT) strawberry (Fig. 2A). Consistently, the crown diameter and plant height of *FvWRKY50* CR 3-16 were significantly lower than WT while *FvWRKY50* CR 2-9 showed no significant difference (Fig. 2B). These results indicated that *FvWRKY50* was involved in the regulation of strawberry plant growth.

In order to explore the regulation function of *FvWRKY50*, RNA sequencing (RNA-seq) experiments were conducted to analysis the transcriptome of WT, *FvWRKY50* CR 2-9, and 3-16 leaves. Analysis results showed that there were 747 differentially expressed genes (DEGs) between WT and *FvWRKY50* CR 2-9, among which 313 were upregulated and 434 were downregulated (Supplementary Data Fig. S2A), while between WT and *FvWRKY50* CR 3-16 there were 2787 DEGs, among which 1483 were upregulated and 1304 were downregulated (Supplementary Data Fig. S2B). Gene ontology (GO) enrichment analysis of the DEGs between WT and *FvWRKY50* CR 3-16 were conducted, which revealed that many DEGs were significantly enriched in biological process related to response to auxin (Supplementary Data Fig. S2C). As the phytohormone auxin plays an important role in plant growth by regulating cell expansion, division, elongation, and differentiation [40], we determined the auxin content and found that auxin content was decreased significantly in both *FvWRKY50* CR 2-9 and 3-16 leaves compared with WT leaves (Fig. 2C). Consistent with these results, transcript levels of *FvYUCCA6* (*FvYUC6*) and *FvYUCCA3* (*FvYUC3*), which catalyze a rate-limiting step in auxin biosynthesis [24], were decreased in *FvWRKY50* CR 2-9 and *FvWRKY50* CR 3-16 plants respectively (Supplementary Data Fig. S3). However, the transcript levels of genes in the auxin signaling pathway were differently regulated between *FvWRKY50* CR 2-9 and *FvWRKY50* CR 3-16. For example, genes for many auxin-responsive proteins, like SAMLL AUXIN UP RNA (SAURs), ARGs, IAAs, auxin transporters (ATLs) and auxin binding proteins (ABPs), were downregulated significantly in *FvWRKY50* CR 3-16 plants relative to WT plants, while the expression levels of most of these genes in *FvWRKY50* CR 2-9 did not decline significantly compared with WT (Fig. 2D). These results suggested that the dramatic changes in genes in the auxin signaling pathway other than the auxin synthesis pathway might cause the dwarf phenotype of *FvWRKY50* CR 3-16 plants.

In addition to auxin-responsive genes, the transcript levels of many other phytohormone genes, like abscisic acid-, jasmonate- and gibberellin-related genes, transcription factor-encoding genes belonging to the *AP2/ERF*, *bZIP*, *bHLH*, *MYB*, *NAC*, and *WRKY* families, and light-responsive genes, were also changed significantly in *FvWRKY50* CR 3-16 plants (Table 1). qRT–PCR results confirmed the changes of some DEGs between WT and *FvWRKY50* CR plants (Fig. 2E). All these results indicated that *FvWRKY50* might play essential roles in diverse biological processes in strawberry.

**Table 1:** Selected DEGs in *FvWRKY50* CR 3-16 leaves compared with WT leaves, and comparison of these genes between WT and *FvWRKY50* CR 2-9

**Functional category**	**Gene ID**	**Annotation**	**CR 3-16 vs WT log**_**2**_ **fold change**	**CR 2-9 vs WT log**_**2**_ **fold change**
Plant hormone				
Auxin	FvH4_2g20150	Indole-3-pyruvate monooxygenase YUCCA3	−2.63	−1.73
	FvH4_7g11280	Auxin responsive protein SAUR36	1.05	0.66
	FvH4_2g10850	Auxin responsive protein ARG7	−2.89	−0.09
	FvH4_5g22810	Auxin responsive protein ARG7	−2.36	−0.53
	FvH4_5g22620	Auxin responsive protein ARG7	−1.66	−0.32
	FvH4_5g22780	Auxin responsive protein ARG7	−1.73	−1.65
	FvH4_6g02870	Auxin-responsive protein IAA4	−1.45	−0.38
	FvH4_4g04700	Auxin-responsive protein IAA13	−1.15	−0.45
	FvH4_6g30850	Auxin-responsive protein IAA17	−1.41	−0.14
	FvH4_2g28970	Auxin-responsive protein IAA29	−2.09	−0.38
	FvH4_4g26700	Auxin-responsive protein IAA32	−3.14	−1.25
	FvH4_2g38760	Auxin response factor 5	1.13	−0.23
	FvH4_3g32000	Auxin response factor 18	−1.79	−0.73
	FvH4_6g30860	Auxin-induced protein AUX22	−2.04	−0.89
Abscisic acid	FvH4_3g16730	9-cis-epoxycarotenoid dioxygenase NCED	1.98	1.54
	FvH4_3g36810	Abscisic acid 8′-hydroxylase 2	2.49	1.50
	FvH4_2g14100	Abscisic acid 8′-hydroxylase 3	−2.30	−0.57
	FvH4_6g53030	β-Glucosidase 13	1.36	−0.11
	FvH4_6g39430	UDP-glucuronosyl and UDP-glucosyl transferase	3.04	0.95
	FvH4_3g16470	Abscisic acid receptor PYL3	−3.29	−2.15
	FvH4_3g07720	Abscisic acid receptor PYL13	−3.12	−3.87
Jasmonate	FvH4_2g40510	Allene oxide synthase 1, AOS1	2.39	0.11
	FvH4_2g07410	Allene oxide synthase 3, AOS3	3.04	0.84
	FvH4_5g32640	Oxophytodienoate reductase 1, OPR1	1.72	0.38
	FvH4_5g32690	NADH oxidase family, OPR2	4.16	1.73
	FvH4_1g08454	NADH oxidase family, OPR3	3.32	2.14
	FvH4_7g00440	Lipoxygenase, LOX	2.85	2.18
	FvH4_2g39441	Jasmonic acid carboxyl methyltransferase 2	1.97	0.77
Gibberellin	FvH4_3g02670	Gibberellin 2-β-dioxygenase 8	1.33	0.91
	FvH4_2g38480	Gibberellin-regulated protein 1	1.02	−0.31
	FvH4_6g48590	Gibberellin-regulated protein 4	−2.63	−0.49
	FvH4_5g14950	Gibberellin-regulated protein 6	−3.01	−1.97
Transcription factors				
AP2/ERF family	FvH4_7g30930	Ethylene-responsive transcription factor CRF3	1.89	1.72
	FvH4_1g03190	Ethylene-responsive transcription factor WIN1	−1.11	−0.49
	FvH4_6g26090	Ethylene-responsive transcription factor 53	1.36	0.56
	FvH4_4g03460	Ethylene-responsive transcription factor 98	−1.11	−0.06
	FvH4_2g13240	Ethylene-responsive transcription factor 110	3.52	−0.73
bZIP family	FvH4_2g36400	bZIP transcription factor 9	−1.47	−0.88
	FvH4_7g25870	bZIP transcription factor 18	−1.05	−1.01
	FvH4_6g26970	bZIP transcription factor 34	−2.3	−1.68
	FvH4_3g24830	bZIP transcription factor TGA	1.25	0.34
bHLH family	FvH4_4g07090	Transcription factor bHLH35	1.61	0.18
	FvH4_7g24720	Transcription factor bHLH51	−1.27	0.10
	FvH4_2g09030	Transcription factor bHLH63	−1.29	−0.61
	FvH4_7g17790	Transcription factor bHLH67	−1.03	−0.65
	FvH4_1g18930	Transcription factor bHLH93	−1.63	0.09
	FvH4_2g39400	Transcription factor bHLH110	1.75	0.52
	FvH4_4g20270	Transcription factor bHLH148	−1.14	−0.95
	FvH4_3g09050	Transcription factor HLH162	−3.85	−0.16
MYB family	FvH4_5g19400	Transcription factor MYB3	−1.62	−2.35
	FvH4_7g16990	MYB-like transcription factor 4	1.74	1.76
	FvH4_4g03610	Transcription factor MYB14	1.99	0.43
	FvH4_6g08620	Transcription factor MYB61	−1.84	−0.75
	FvH4_5g11930	Transcription factor MYB62	3.12	1.65
	FvH4_1g02690	Transcription factor MYB82	−3.62	−0.39
	FvH4_2g31100	Transcription factor MYB123-like	2.43	2.34
NAC family	FvH4_3g20700	NAC transcription factor RIF	1.68	−0.74
	FvH4_2g16180	NAC transcription factor 6	1.55	0.53
	FvH4_6g51660	NAC transcription factor 8	1.72	0.70
	FvH4_6g33050	NAC transcription factor 35	−1.21	−0.12
	FvH4_5g21670	NAC transcription factor 71	1.03	1.11
	FvH4_6g19430	NAC transcription factor 90	1.73	0.46
WRKY family	FvH4_1g16480	WRKY transcription factor 15	1.24	−0.09
	FvH4_3g39850	WRKY transcription factor 24	1.15	0.02
	FvH4_2g41070	WRKY transcription factor 40	3.35	0.52
	FvH4_1g26200	WRKY transcription factor 42	1.87	−0.54
	FvH4_2g31400	WRKY transcription factor 50	2.70	0.92
	FvH4_5g15340	WRKY transcription factor 72	2.42	0.97
Light responsive	FvH4_4g22790	Light-regulated protein 1	−2.06	−0.39
	FvH4_2g20400	Early light-induced protein 1	1.51	2.71
	FvH4_7g15980	Light-sensor protein kinase PHY1	1.07	0.17
	FvH4_6g02940	Light-inducible protein CPRF2	−1.73	−1.03
	FvH4_7g26541	Light-harvesting complex-like protein 3	1.36	2.21

### *FvWRKY50* regulated strawberry leaf senescence by directly targeting *SAUR36*

*SAUR* (small auxin up RNA) genes have been reported to be the key regulators of leaf senescence [12, 37]. RNA-seq data and qRT–PCR results showed that *SAUR36* and *SAUR72* were significantly upregulated in *FvWRKY50* CR 2-9 and 3-16 plants (Fig. 2D and E). Further correlation analysis showed that *FvWRKY50* was co-expressed with *SAUR36* and other senescence-related genes, including *senescence-associated carboxylesterase 101* (*SG101*), *senescence-specific cysteine protease 39* (*SAG39*), *senescence-associated transcription factor JUB1*, *WRKY70* [15, 16] and so on (Supplementary Data Fig. S4A; Supplementary Data Table S1). These results suggested that *FvWRKY50* might be involved in leaf senescence in strawberry.

**Figure 3:**
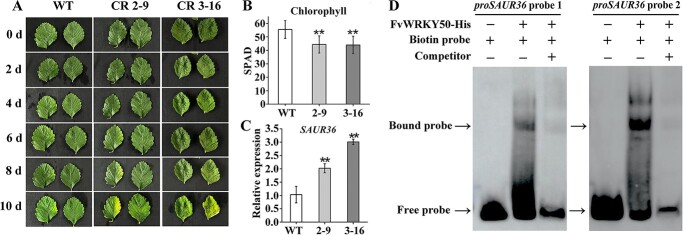
. *FvWRKY50* regulated strawberry leaf senescence by directly targeting the *SAUR36* promoter. (A) Leaf senescence progress of WT, *FvWRKY50* CR 2-9, and *FvWRKY50* CR 3-16 leaves. Detached leaves of WT and *FvWRKY50* CR plants were placed in Petri dishes with wet filter paper to observe leaf senescence phenotypes and were photographed every 2 days. (B) Chlorophyll content of detached leaves of WT, *FvWRKY50* CR 2-9, and *FvWRKY50* CR 3-16. A chlorophyll meter was used to determine chlorophyll content 10 days after detachment. (C) Expression level of *SAUR36* in WT, *FvWRKY50* CR 2-9, and *FvWRKY50* CR 3-16 leaves at 10 days after detachment. qRT–PCR assays were used in this experiment. (D) EMSA assays showing the direct binding of FvWRKY50 to the *SAUR36* promoter. The *proSAUR36* probes 1 and 2 containing W-box were used as biotin probes. Unlabeled probes (200-fold excess) were used as competitors.

We then used detached leaves from WT, *FvWRKY50* CR 2-9, and 3-16 lines to observe the process of leaf senescence. At 8 days after detachment *FvWRKY50* CR 2*-*9 and 3-16 leaves began to turn yellow while WT leaves were still green, indicating that *FvWRKY50* CR leaves initiated senescence more quickly than WT leaves. At 10 days after detachment the WT leaves began to turn yellow (Fig. 3A). We determined the chlorophyll contents at 10 days, and the results showed that chlorophyll content decreased more rapidly in *FvWRKY50* CR leaves than WT leaves (Fig. 3B). The expression level of *SAUR36* was significantly increased in *FvWRKY50* CR plants compared with WT (Fig. 3C). These results indicated that *FvWRKY50* regulated leaf senescence in strawberry, possibly by modulating the expression of senescence-related genes.

WRKY transcription factors regulate target genes by binding to the W-box elements of target gene promoter sequences. To determine whether FvWRKY50 could regulate senescence-related genes directly, we analyzed the promoter sequences of these genes and found there were two W-box elements at 1483 and 1636 bp upstream of ATG in the *SAUR36* promoter. Electrophoretic mobility shift assay (EMSA) results showed that the recombinant FvWRKY50-His fusion protein could bind to the DNA probes containing the W-box motif 1 and 2 of the *SAUR36* promoter, and the unlabeled probe could compete this binding effectively (Fig. 3D). These results suggested that *FvWRKY50* negatively regulated strawberry leaf senescence by directly targeting senescence-related genes like *SAUR36* to modulate their expression level.

### *FvWRKY50* mutation affected flowering time of strawberry by regulating expression of *FvFT2*, *FvFT3*, and *FvCO*

**Figure 4:**
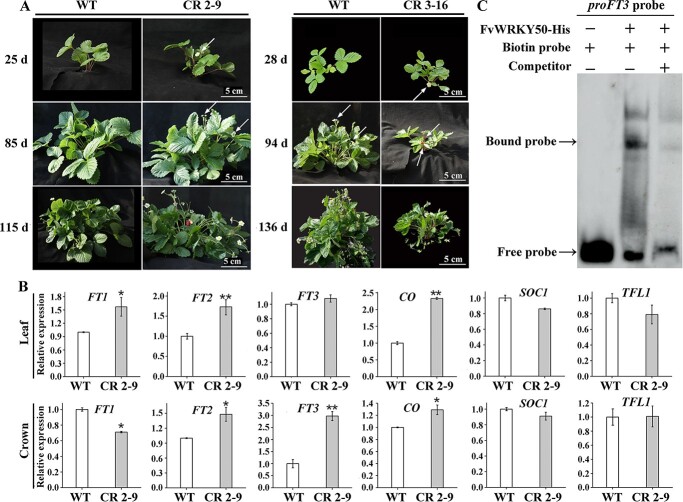
*FvWRKY50* mutation influenced flowering time of strawberry plant. (A) Phenotypes of WT and *FvWRKY50* CR plants at different developmental stages. WT and *FvWRKY50* CR 2-9 plant phenotypes were observed and photographed 25, 85, and 115 days after planting. WT and *FvWRKY50* CR 3-16 plant phenotypes were observed and photographed 28, 94 and 136 days after planting. Flowers and fruits at 25, 85, 28 and 94 days are indicated by white arrows. (B) Relative expression levels of flowering-related genes in WT and *FvWRKY50* CR 2-9. qRT–PCR assay was used to analyze the expression levels of *FT1*, *FT2*, *FT3*, *CO*, *SOC1*, and *TFL1* in leaves and crown of WT and *FvWRKY50* CR 2-9. (C) EMSA assays showing that FvWRKY50 could bind to the *FT3* promoter directly. *proFT3* probes containing W-box were used as biotin probes. Unlabeled probes (200-fold excess) were used as competitors.

By continuous phenotype observation we found that the *FvWRKY50* mutation induced an early flowering phenotype compared with WT plants. It took ~30 days for *FvWRKY50* 2-9 and 3-16 plants to come into the anthesis stage while for WT it took ~100 days (Fig. 4A). Previous studies have identified flowering-related genes in strawberry, including florigen *FTs*, *TFL1*, *CO*, and *SOC1* [8, 28, 47]. We examined the expression of *FvFT1*/*2*/*3*, *FvCO*, *FvSOC1*, and *FvTFL1* in leaf and crown. qRT–PCR results showed that the transcript levels of positive flowering regulators *FvFT1*, *FvFT2*, and *FvCO* were higher in *FvWRKY50* CR 2-9 leaf than in WT leaf, while in the crown *FvFT3* gene expression was higher in *FvWRKY50* CR 2-9 than in WT (Fig. 4B). Promoter motif analysis showed that there were W-box motifs in the promoter sequence of *FT3*, and EMSA results showed that the recombinant FvWRKY50-His fusion protein could bind to a DNA probe containing the W-box motif of the *FT3* promoter (Fig. 4C). These results suggested that FvWRKY50 played a negatively role in strawberry flowering by directly or indirectly regulating *FT* and *CO* gene expressions.

### *FvWRKY50* was involved in the regulation of fruit ripening and anthocyanin accumulation in strawberry fruit

**Figure 5:**
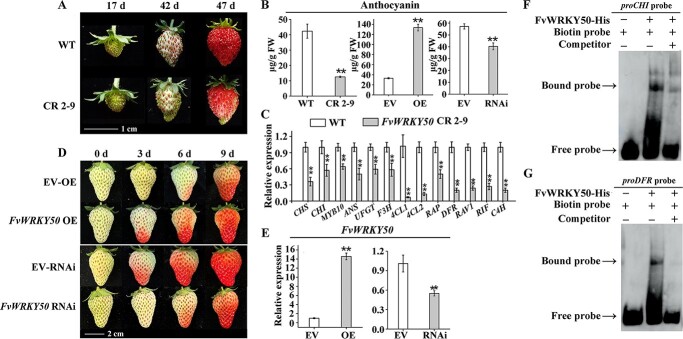
. *FvWRKY50* regulated anthocyanin accumulation in strawberry fruit*.* (A) Anthocyanin accumulation phenotypes of WT and *FvWRKY50* CR 2-9 fruits at different developmental stages. Fruits of WT and *FvWRKY50* CR 2-9 were harvested and photographed 17, 42, and 47 days after anthesis. (B) Anthocyanin content of WT and *FvWRKY50* CR 2-9 (left), EV-OE, and transient OE (middle), and EV-RNAi and transient RNAi (right) fruits. (C) Anthocyanin accumulation-related gene expression levels of WT and *FvWRKY50* CR 2-9 fruits 47 days after anthesis. (D) Phenotypes of *FvWRKY50-*OE and *FvWRKY50-*RNAi fruits. Octoploid strawberry fruits at big green stage were selected for transient transformation. Control (EV-OE, EV-RNAi) and transient (*FvWRKY50*-OE, *FvWRKY50*-RNAi) fruits were photographed 0, 3, 6 and 9 days after injection. (E) *FvWRKY50* gene expression levels in EV-OE and *FvWRKY50*-OE (transient OE) and EV-RNAi, *FvWRKY50*-RNAi (transient RNAi). qRT–PCR assay was used. (F, G) EMSAs showing that FvWRKY50-His protein could bind to *CHI* (F) and *DFR* (G) promoters directly. Negative control is shown in the first lane. Unlabeled probe (200-fold excess) was used as competitor.

To evaluate the effects of *FvWRKY50* mutation on strawberry fruit ripening, we observed the fruit phenotype of *FvWRKY50* CR and WT plants. As homozygous mutation of *FvWRKY50* made the fruits of *FvWRKY50* CR 3-16 too small and severely deformed, which might affect observations (Supplementary Data Fig. S5), *FvWRKY50* CR 2-9 and WT fruit phenotypes at different developmental stages were observed. We found that anthocyanin accumulation of *FvWRKY50* CR 2-9 fruits was delayed compared with WT fruits (Fig. 5A), consistent with the lower anthocyanin content in *FvWRKY50* CR 2-9 fruits (Fig. 5B). The expression levels of anthocyanin accumulation-related genes were determined by qRT–PCR and results showed that *CHS*, *CHI*, *MYB10*, *ANS*, *UFGT*, *F3H*, *4CL1*, *4CL2*, *RAP*, *DFR*, *RAV1*, *RIF*, and *C4H* were all downregulated significantly in *FvWRKY50* CR 2-9 fruit compared with WT (Fig. 5C). Since RIF is also a key transcription factor affecting the formation of sugar, acid, and other qualities of strawberry fruit, FvWRKY50 may play a more important role rather than specifically regulating anthocyanin accumulation. We transiently overexpressed *FvWRKY50* (*FvWRKY50*-OE) and knocked down *FvWRKY50* (*FvWRKY50-*RNAi) in cultivated strawberry fruits. We observed an acceleration in anthocyanin accumulation in *FvWRKY50*-OE fruits and delayed anthocyanin accumulation of *FvWRKY50*-RNAi fruits during the ripening process compared with WT fruits (Fig. 5B and D). qRT–PCR results validated the overexpression and downregulation expression level of *FvWRKY50* in *FvWRKY50*-OE and *FvWRKY50-*RNAi fruits respectively (Fig. 5E). Furthermore, EMSA results showed that FvWRKY50 could regulate anthocyanin accumulation in strawberry fruits by directly regulating *FvCHI* and *FvDFR* gene expression (Fig. 5F and G). These results showed that FvWRKY50 was an important regulator of fruit ripening, especially in anthocyanin accumulation.

### FvWRKY50 was phosphorylated and degraded by FvMAPK3 under low temperature

**Figure 6:**
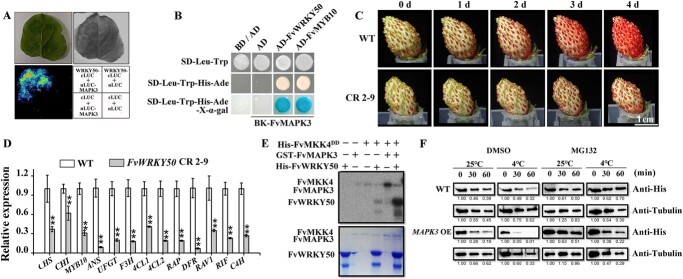
FvMAPK3 phosphorylated and degraded FvWRKY50 protein under low temperature to affect anthocyanin accumulation in strawberry fruit*.* (A) Luciferase complementary imaging assays showing the interaction between FvWRKY50 and FvMAPK3 *in vivo*. Various constructs were co-infiltrated into *N. benthamiana* leaves, and LUC activity was captured. (B) Y2H assays showing the interaction between FvWRKY50 and FvMAPK3. The interaction between FvMYB10 and FvMAPK3 was the positive control. (C) Anthocyanin accumulation phenotypes of WT and *FvWRKY50* CR 2-9 fruits at 10°C for low temperature treatment. White stage fruits of WT and *FvWRKY50* CR 2-9 were harvested and cultured in 88 mM sucrose solution at 10°C. Fruits were photographed at different days after treatment. (D) Anthocyanin accumulation-related gene expression of WT and *FvWRKY50* CR 2-9 fruits at 4 days after low temperature treatment. (E) FvMAPK3 phosphorylated FvWRKY50 *in vitro*. His-FvMKK4^DD^, GST-FvMAPK3, and His-FvWRKY50 recombinant proteins were incubated in kinase reaction buffer and then detected by SDS–PAGE. The autoradiograph (top) and CBB staining (bottom) of the proteins are indicated. (F) Cell-free degradation assay of recombinant His-FvWRKY50. Total proteins of WT and *FvMAPK3*-OE fruits were extracted and incubated with recombinant His-FvWRKY50 protein at 25 and 4°C with 50 mM MG132 or DMSO. His-FvWRKY50 protein was detected with anti-His antibody and tubulin was detected with anti-tubulin.

To reveal the molecular mechanism of *FvWRKY50* in regulating strawberry fruit ripening, we screened the interacting proteins of FvWRKY50. Bimolecular fluorescence complementation (BiFC) and yeast two-hybrid (Y2H) results both indicated that FvWRKY50 interacted with strawberry MITOGEN-ACTIVATED PROTEIN KINASE3 (FvMAPK3; Fig. 6A and B). FvMAPK3 was reported as an important negative regulator of anthocyanin accumulation in strawberry fruits and could be activated by low temperature to repress anthocyanin accumulation [26].

To evaluate whether FvWRKY50 was involved in low temperature-repressed anthocyanin accumulation, we examined the effects of 10°C treatment on anthocyanin accumulation in WT and *FvWRKY50* CR 2-9 fruits. As shown in Fig. 6C, the accumulation of anthocyanin in *FvWRKY50* CR 2-9 fruits was significantly delayed compared with that in WT fruits, and accordingly the anthocyanin accumulation-related genes were all downregulated significantly (Fig. 6D). These results indicated that *FvWRKY50* CR 2-9 fruit was more sensitive to low temperature and FvWRKY50 acted as a downstream component of low temperature signal during anthocyanin accumulation in strawberry fruit.

To test whether FvWRKY50 was involved in FvMAPK3-mediated anthocyanin accumulation as its phosphorylation substrate, we conducted an *in vitro* kinase assay using GST-FvMAPK3 protein and His-FvWRKY50 protein. The results showed that FvMAPK3 was activated by His-FvMKK4^DD^ and then phosphorylated His-FvWRKY50, indicating that FvWRKY50 was the phosphorylation substrate of FvMAPK3 (Fig. 6E). In addition, the proteasome inhibitor MG132 decelerated the FvMAPK3-mediated degradation of FvWRKY50, which suggested that the degradation of FvWRKY50 was regulated by 26S proteasome-mediated ubiquitination (Fig. 6F). These results indicated that FvWRKY50 was an important downstream regulator of FvMAPK3 in low temperature-mediated anthocyanin accumulation in strawberry fruits.

**Figure 7:**
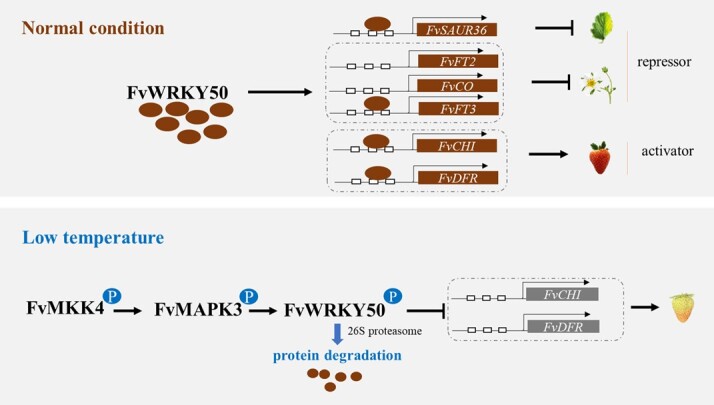
*FvWRKY50* is an important gene that regulates both vegetative growth and reproductive growth in strawberry. During vegetative growth, FvWRKY50 negatively regulates leaf senescence by directly targeting *FvSAUR36*. Then FvWRKY50 affects flowering time mainly by repression the expression of *FvFT2*, *FvFT3*, and *FvCO*. During the fruiting stages, complete mutation of *FvWRKY50* leads to abnormal fertility of strawberry. High expression of *FvWRKY50* can promote ripening and coloring of strawberry fruit. However, at low temperature FvWRKY50 is phosphorylated by FvMAPK3, and ubiquitination-mediated degradation of FvWRKY50 protein occurs, resulting in delayed fruit coloring. In conclusion, *FvWRKY50* is a key gene in the regulation of strawberry growth and fruit ripening. It is of great value to continue to explore the regulatory mechanisms of *FvWRKY50* in different biological processes of strawberry.

In conclusion, *FvWRKY50* is an important gene regulating vegetative and reproductive growth of strawberry, and plays a key role in strawberry fruit response to low temperature (Fig. 7). The identification of this gene provides valuable clues to reveal the molecular regulation mechanism of strawberry growth and fruit ripening, especially the regulation of plant growth and fruit ripening by low temperature. The creation of *FvWRKY50* mutants, especially the biallelic mutant, could lay an important foundation for improving strawberry flowering time by genome editing technology.

## Discussion

WRKY transcription factors belong to a large gene family and have diverse functions [32,55]. However, relative to other species the regulatory function of specific WRKY transcription factors in strawberry is little known. In this study we identified a WRKY transcription factor, FvWRKY50, in diploid strawberry. Phylogenetic analysis showed that FvWRKY50 was close to RcWRKY45 and AtWRKY75 in genetic relationship (Fig. 1A). The function of RcWRKY45 has not been clear, while several studies have indicated that AtWRKY75 functions in multiple physiological processes, including nutrient starvation response, root development, leaf senescence, and flowering [6, 9, 33, 50]. Our findings suggest that FvWRKY50 regulated strawberry leaf senescence and flowering negatively while regulating strawberry fruit ripening and anthocyanin accumulation positively (Figs 2–5). Interestingly, FvWRKY50 had no transcriptional activation activity (Fig. 1C). It is speculated that FvWRKY50 can recruit different transcription factors to form a transcription complex and act as a transcriptional activator or repressor, which may be an important reason for the diverse functions of FvWRKY50.

Although only *FvWRKY50*-homozygous plants dwarfed and had smaller plant size, there was no significant difference in the decrease in auxin content between biallelic and homozygous mutants, indicating that the difference did not result from the change in auxin content (Fig. 2A and C). Transcriptome data analysis and qRT–PCR results both revealed that many auxin-related gene transcript levels were more significantly downregulated in *FvWRKY50* CR 3-16 plants, including auxin transporters *ATL2*/*3*, auxin binding proteins (*ABP*s), and auxin-responsive protein genes like *IAA*s and *ARG*s (Fig. 2D). Based on the above results, we speculated that the differences in plant type between the two mutants might be associated with their sensitivity to auxin or not be related to auxin.

*SAUR* is an important gene family that has attracted much attention in recent years. In addition to participating in auxin-mediated plant development, SAUR can also play its biological functions independently of auxin [37]. However, little is known about *SAURs* in strawberry. In this study, we found that the expression of three *SAUR* genes, *SAUR36*, *SAUR72*, and *SAUR50*, was significantly regulated in *FvWRKY50* mutants and that FvWRKY50 bound the promoter of *SAUR36* (Fig. 3). The homolog gene of *SAUR36* has been demonstrated as an important regulator of leaf senescence, and we found that *SARU36* was much more significantly upregulated in senescent leaves of *FvWRKY50* mutants [12] (Fig. 3A). Our finding validates that *SAUR36* can conservatively regulate leaf senescence in *A. thaliana* and strawberry, and can also provide valuable clues for further revealing the function of *SAUR* genes such as *SARU72* and *SAUR50* in strawberry.

Besides vegetative growth, *FvWRKY50* was also involved in the regulation of reproductive growth, including flowering and fruit ripening (Figs 4 and 5). Flowering time is an important economic character of strawberry which determines the ripening period. Previous studies have identified a few important structural genes involved in strawberry flowering using transgenic materials, including *FvFT1*, *FvFT2*, *FvFT3*, *FvCO*, *FvSOC1*, and *FvTFL1* [8, 20, 28, 31]. However, the transcriptional regulatory mechanism of these genes is not clear. In this study, we demonstrated that FvWRKY50 bound the promoter of *FvFT3* and significantly regulated the expression of *FvFT*s and *FvCO* in strawberry crown (Fig. 4). Considering that overexpressed *FvFT3* regulated strawberry plant branching and *FvWRKY50* homozygous mutant showed smaller plant size, FvWRKY50 may also function in the regulation of strawberry plant morphology [8]. In addition to FvWRKY50, FvWRKY71 has also been reported to be involved in flowering regulation by affecting the expression of *FvFUL* and *FvLFY* [22], suggesting that different *WRKY* genes may cooperatively regulate strawberry flowering by regulating different flowering structure genes. Our results also showed that other *WRKY* genes, such as *FvWRKY53* and *FvWRKY70*, were significantly regulated in *FvWRKY50* mutants (Fig. 2). The function of other FvWRKYs in flowering regulation needs to be examined in the future. Notably, overexpressed *FvYUC6* or *FvARF4* delayed or promoted flowering, suggesting that auxin may be the important hormone involved in flowering regulation in strawberry in a concentration-dependent or sensitive-dependent manner [7, 24]. Given that FvWRKY50 was highly associated with auxin biosynthesis and signaling transduction, whether and how FvWRKY50 participates in auxin-mediated flowering is worth further exploration.

While FvWRKY50 negatively regulated plant growth, flowering time, and leaf senescence, it regulated anthocyanin accumulation positively in strawberry fruit (Fig. 5). Besides structural genes, the transcription factor genes related to anthocyanin accumulation and fruit ripening, such as *MYB10*, *RAP*, *RAV*, and *RIF*, were also significantly regulated by FvWRKY50, suggesting that FvWRKY50 may be involved in the regulation of fruit ripening upstream of these important transcription factors. The specific roles of FvWRKY50 in fruit ripening and the formation of other quality indexes is worth further study. Additionally, the FvWRKY50 homozygous mutant hardly bore normal fruits, indicating that FvWRKY50 was also involved in the regulation of strawberry fertility, which may affect the pollination and fertilization process (Supplementary Data Fig. S5).

Low temperature is one of the important environmental factors affecting fruit ripening, especially anthocyanin accumulation, in strawberry fruit [26]. However, little is known about how low temperature affects anthocyanin accumulation in strawberry fruits. Recently, we revealed that protein kinase FvMAPK3 was the critical regulator mediating low-temperature repression of anthocyanin accumulation by phosphorylating FvMYB10 and FvCHS1 [26]. In this study, we found that FvWRKY50 acted as the substrate of FvMAPK3 in low-temperature repression of anthocyanin accumulation in strawberry fruit (Figs 5 and 6). FvMAPK3 could induce the degradation by phosphorylating FvWRKY50 (Fig. 6). Previous studies have demonstrated that different members of MAPK and WRKY can form various phosphorylation modules to fine-regulate disease resistance signals in *A. thaliana* and rice [30, 40]. Considering the multiple regulatory functions of FvWRKY50, further exploration of whether WRKY50 can form phosphorylation modules with other MAPKs will provide clues for the identification of new genes regulating strawberry development and fruit ripening, and lay a foundation for further revealing the post-transcriptional regulatory mechanism of strawberry development and fruit ripening. Additionally, given the important roles of low temperature in the regulation of flowering and fruit ripening, it is necessary to further clarify whether other biological functions of FvWRKY50 are also related to its involvement in low temperature response.

## Materials and methods

### Plant materials and growing conditions

In this study diploid strawberry *Fragaria vesca* cultivar ‘Fragola di Bosco’ and octoploid strawberry *F.* × *ananassa* Duch. cultivar ‘Benihoppe’ were used. Strawberry plants were grown under controlled photoperiod (12 hours light/12 hours dark) with a light intensity of 200–300 μmol m^−2^ s^−1^ (white fluorescent tube, T5, 14 W). The temperature was 25°C in the daytime and 15°C at night, and the relative humidity was controlled at 70%.

After rooting in tissue culture bottles, the transgenic plants were transplanted into 10-cm seedling pots. After 1 month of culture, the transgenic plants were transplanted together with WT into large white pots and filed for phenotype observation.

### Low temperature treatment

To observe the effects of low temperature on anthocyanin accumulation, detached white fruits of WT and *FvWRKY50* CR 2-9 were cultured in 88 mM sucrose solution at 10°C under a light intensity of 50 μmol m^−2^ s^−1^ in a growth chamber. Samples were frozen in liquid nitrogen after 4 days of treatment, and then stored at −80°C for anthocyanin determination and qRT–PCR analysis.

To analyze the effects of low temperature-induced phosphorylation of FvMAPK3 on FvWRKY50 protein stability, white fruits of WT and *FvMAPK3* OE were treated at 4°C and 25°C for 1 hour. Samples were frozen in liquid nitrogen after treatment, and then stored at −80°C for protein extraction.

### Leaf senescence phenotype observation

Mature leaves of WT, *FvWRKY50* CR 2-9, and *FvWRKY50* CR 3*-*16 were detached and placed in Petri dishes with wet filter paper to observe the progress of leaf senescence. The Petri dishes were wrapped in plastic film to keep the high humidity. The leaves were photographed every 2 days and samples were frozen in liquid nitrogen 10 days after detachment.

### Transient transformation for strawberry fruits

To transiently overexpress *FvWRKY50* in strawberry fruits, the coding sequence of *FvWRKY50* was cloned from strawberry cDNA and reconstructed into pH7WG2D vector using the Gateway method. To construct the *FvWRKY50* RNAi plasmid, two parts of the *FvWRKY50* coding sequence were cloned into pFGC5941 vector in opposite orientation. *Agrobacterium* EHA105 was used to transfect the fruit cells. When the *Agrobacterium* culture had reached OD_600_ 0.6–0.8, the bacterial solution was centrifuged at 25°C for 10 minutes at 5000 g and then suspended in a buffer solution containing 10 mM MES pH 5.6, 10 mM MgCl_2_, and 200 μM acetosyringone, and shaken at 25°C for 1 hour.

In the transient transformation experiments, bacterial suspensions of EV-OE, *FvWRKY50*-OE, EV-RNAi or *FvWRKY50*-RNAi were injected into the octoploid strawberry fruits at the big green stage with 1-ml syringes, and 20 fruits were treated each time for biological repeats. The strawberry fruit was continuously photographed. The fruits were harvested 9 days after injection, frozen in liquid nitrogen, and stored at −80°C for subsequent gene expression analysis and anthocyanin detection.

### Stable transformation of diploid strawberry

Genome editing of *FvWRKY50* was performed using the CRISPR/Cas9 system, and two sgRNAs were constructed in the coding sequence region of *FvWRKY50* and cloned into pYLCRISPR/Cas9 vector. Subsequently, the constructed *FvWRKY50* CR vector was transformed into diploid strawberry as described previously [29]. After screening in medium containing 2 mg/l hygromycin, DNA was extracted from strawberry seedlings with hygromycin resistance and sequenced to validate the editing method. The specific primers listed in Supplementary Data Table S2 were used for sequencing analysis of target genes by PCR. WT plants and *FvWRKY50* mutants were subcultured at the same time, and then transplanted into the growth chamber or greenhouse according to the same procedure. At least three plants of each edited type were used for phenotype analysis.

### Yeast two-hybrid assay

A GAL4-based Two-Hybrid System 3 (Clontech, Mountain View, CA, USA) was selected to conduct the Y2H assay. The *FvWRKY50* coding sequence was cloned into pGADT7 vector and the FvMAPK3 coding sequence was cloned into pGBKT7 vector. The plasmids were co-transformed into yeast strain AH109 and cultured in −Leu−Trp and −Leu−Trp−His−Ade medium. After 3 days of culture, the colonies were stained with X-α-gal. Primers used for cloning are listed in Supplementary Data Table S2.

### Luciferase complementary imaging assay

The full-length coding sequences of *FvWRKY50* and *FvMAPK3* were cloned into pCAMBIA1300-cLUC vector and pCAMBIA1300-nLUC vector, respectively. Then the nLUC or cLUC construct was transformed into *Agrobacterium* strain EHA105 and the bacterial solutions were co-transformed at a 1:1 ratio into *N. benthamiana* leaves to observe the luciferase activity. Primers used for cloning are listed in Supplementary Data Table S2.

### Protein subcellular localization

The coding sequence region of *FvWRKY50* was cloned into the pSuper-GFP vector, and the constructed vector was transformed into *Agrobacterium* strain GV3101. Buffer solution with *Agrobacterium tumefaciens* was infiltrated into *N. benthamiana* leaves at the seedling age of 50 days. After 2–3 days, tobacco epidermal cells were observed using a confocal laser scanning microscope (Leica SP8). Primers used for constructs are listed in Supplementary Data Table S2.

### Recombinant protein production and purification

Full-length cDNAs of *FvWRKY50*, *FvMAPK3*, and *FvMAPKK4* were amplified and connected to the vectors pET30a, pGEX4T1, and pET30a respectively to generate the vectors His-FvWRKY50, GST-FvMAPK3, and His-FvMAPKK4 (FvMKK4^DD^, activated state of FvMKK4). The plasmids were then transformed into *Escherichia coli* BL21 (DE3) cells. Recombinant proteins of His-FvWRKY50, GST-FvMAPK3, and His-FvMAPKK4 were purified using glutathione agarose beads (GE Healthcare) and Ni-NTA agarose (Novagen).

### Plant protein extraction and western blotting

Strawberry fruits were ground into powder in liquid nitrogen and then total proteins were extracted with extraction buffer [phosphate buffers pH 7.8, 1 mM EDTA, 10% (v/v) glycerol, 0.5% (v/v) Triton X-100, 1 mM DTT, 1 mM benzoyl sulfonyl fluoride, 1 × protease inhibitor mixture, and 1 × phosphatase inhibitor mixture]. Then, the homogenate was centrifuged at 13 000 g for 10 minutes, and the supernatant was analyzed using 10% sodium dodecyl sulfate–polyacrylamide gel electrophoresis (SDS–PAGE). The antibodies anti-His (1:1000, CWBIO) and anti-tubulin (1:2000; Abmart, Shanghai, China) were used for western blotting.

### *In vitro* phosphorylation analysis

Recombinant GST-FvMAPK3 was activated by His-MAPKK4 (FvMKK4^DD^) proteins at 30°C for 30 minutes in buffer (20 mM Tris–HCl at pH 7.5, 10 mM MgCl_2_, and 50 μM ATP). His-FvWRKY50 recombinant protein was then combined with activated GST-FvMAPK3 in kinase reaction buffer [20 mM Tris–HCl at pH 7.5, 10 mM MgCl_2_ 25 μM ATP, and 1 μCi (γ-32p) ATP]. Then the reaction was conducted at 30°C for 30 minutes. The sample was then subjected to 10% SDS–PAGE to isolate the protein. The gel was exposed to a phosphor image screen for 12–24 hours and observed with a Typhoon 9410 imager.

### Anthocyanin content determination

Anthocyanin was extracted using a plant anthocyanin content kit (Kemingshengwu, Suzhou, China). To 0.1 g of sample was added 1 ml of extract liquid, and ultrasonic extraction was performed for 2 hours followed by centrifugation at 8000 g for 10 min, and the supernatant was then analyzed. The content of anthocyanins was determined by the pH differential method.

### RNA-seq analysis

Total RNA was extracted from WT, *FvWRKY50* CR 2-9, and *FvWRKY50* CR 3-16 mature leaves of the same seedling age using an E.Z.N.A. Total RNA Kit (Omega, Bienne, Switzerland). Sequencing was performed on an Illumina Novaseq platform and mapped by using the *F. vesca* subsp. *vesca* reference genome (https://www.rosaceae.org/rosaceae_downloads/Fragaria_vesca/Fvesca-genome.v4.0.a1/assembly/Fragaria_vesca_v4.0.a1.fasta.gz). Based on DESeq2 analysis, |log_2_ fold change| >1 and padj < 0.05 was considered as differential expression. Three independent biological replicates were analyzed for this experiment.

### Real-time quantitative PCR

Following the instrument instructions, qRT–PCR was performed using Taq Pro Universal SYBR qPCR Master Mix (q712 Vazyme, Nanjing China) on a fluorescence quantitative PCR instrument (QuantStudio 6 Flex, Applied Biosystems, Thermo Fisher Scientific, USA). The internal reference was *FvActin* and the relative expression was analyzed by the 2^-ΔΔCT^ method. Three biological replicates were analyzed for the qRT–PCR experiments. Primer sequences used for RT–qPCR are shown in Supplementary Data Table S2.

### Cell-free degradation assays

Total proteins of WT and *FvMAPK3*-OE fruits after low temperature treatment were extracted and incubated with recombinant His-FvWRKY50 protein in buffer (50 mM Tris-MES pH 8.0, 500 mM sucrose, 1 mM MgCl_2_, 10 mM EDTA, and 5 mM DTT). For one treatment, 50 μM MG132 was added to the mixture to inhibit protein degradation while for the other treatment an equal amount of DMSO as control was added into the sample. Both samples were incubated at 37°C and a 30-μl aliquot was taken at 0, 30, and 60 minutes. His-FvWRKY50 protein was detected by western blotting using anti-His antibody.

### Sequence analysis and phylogenetic analysis

ClustalX version 2.1 was used for protein sequence alignments. The phylogenetic tree was constructed by using the neighbor-joining method in MEGA version 5.0 software. The protein sequences of FvWRKY50, RcWRKY45 (XP_024181838.1), AtWRKY75 (NP_196812.1), NtWRKY75 (XP_016446514.1), OsWRKY72 (ALB35168.1), and SlWRKY45 (XP_004233585.1) were used to construct the phylogenetic tree.

## Supplementary Material

Web_Material_uhad115

## Data Availability

Data supporting the results reported in the paper are available in the main text and supplementary data.
