# Supplementary material for: FvWRKY50 is an important gene that regulates both vegetative growth and reproductive growth in strawberry
Source: Hortic Res. 2023 May 31;10(7):uhad115. doi: 10.1093/hr/uhad115 (PMC10419500; doi:10.1093/hr/uhad115)
Supplement: Web_Material_uhad115 [file web_material_uhad115.zip › Table S1-Genes used for co expression analysis.docx]

**Table S1 Selected differential expressed genes (DEGs) in *FvWRKY50* CR 3-16 leaves compared to WT leaves used for the co-expression analysis.**

| Annotation | Functional category | Gene ID | Log_2_FC | Padj |
| --- | --- | --- | --- | --- |
| SAUR36 | Auxin-responsive protein | FvH4_7g11280 | 1.05 | 4.37E-20 |
| SAUR50 | Auxin-responsive protein | FvH4_2g10770 | -1.1 | 1.41E-12 |
| SAUR72 | Auxin-responsive protein | FvH4_3g15390 | 3.36 | 0.0056 |
| YUC3 | indole-3-pyruvate monooxygenase | FvH4_2g20150 | -2.63 | 1.48E-09 |
| SG101-1 | Senescence-associated carboxylesterase 101 | FvH4_2g18461 | 1.42 | 0.0209 |
| SG101-2 | Senescence-associated carboxylesterase 101 | FvH4_6g50162 | 1.23 | 0.0006 |
| SAG39 | Senescence-specific cysteine protease | FvH4_3g14350 | 1.93 | 7.08E-06 |
| EIN3 | ETHYLENE INSENSITIVE 3 | FvH4_7g04852 | 1.33 | 6.56E-05 |
| ARF5 | Auxin response factor | FvH4_2g38760 | 1.13 | 0.0190 |
| bHLH39 | Transcription factor bHLH | FvH4_2g11650 | 2.41 | 2.82E-39 |
| bHLH162 | Transcription factor bHLH | FvH4_3g09050 | -3.85 | 2.67E-05 |
| FaMYB44.1 | Transcription factor MYB | FvH4_1g16770 | 1.16 | 7.17E-19 |
| MYB82 | Transcription factor MYB | FvH4_1g02690 | -3.62 | 4.35E-08 |
| MYB123 | Transcription factor MYB | FvH4_2g36950 | -2.67 | 6.22E-09 |
| NAC2 | Transcription factor NAC | FvH4_2g27430 | 1.52 | 2.01E-23 |
| NAC22 | Transcription factor NAC | FvH4_3g16040 | -2.24 | 9.32E-23 |
| NAC96 | Transcription factor NAC | FvH4_7g18250 | 1.55 | 0.0199 |
| JUB1 | Transcription factor NAC | FvH4_7g01370 | -1.95 | 1.78E-58 |
| WRKY6 | Transcription factor WRKY | FvH4_3g01700 | 2.08 | 1.13E-18 |
| WRKY53 | Transcription factor WRKY | FvH4_7g31050 | 1.30 | 0.0483 |
| WRKY70 | Transcription factor WRKY | FvH4_6g09750 | 1.36 | 1.11E-11 |
| ERF2 | Transcription factor AP2/ERF | FvH4_7g26920 | 1.19 | 4.18E-11 |
| ERF53 | Transcription factor AP2/ERF | FvH4_6g26090 | 1.36 | 0.0007 |
| DREB1B | Transcription factor AP2/ERF | FvH4_6g18090 | 3.68 | 1.79E-10 |
| bZIP29 | Transcription factor bZIP | FvH4_2g38831 | 1.30 | 2.44E-22 |
| bZIP44 | Transcription factor bZIP | FvH4_2g39350 | 1.84 | 2.00E-43 |
| bZIP53 | Transcription factor bZIP | FvH4_1g12620 | -2.80 | 1.01E-18 |
