# Supplementary material for: FvWRKY50 is an important gene that regulates both vegetative growth and reproductive growth in strawberry
Source: Hortic Res. 2023 May 31;10(7):uhad115. doi: 10.1093/hr/uhad115 (PMC10419500; doi:10.1093/hr/uhad115)
Supplement: Web_Material_uhad115 [file web_material_uhad115.zip › Table S2-Primers used in this study.docx]

**Table S2 Primers used in this study.**

| Purpose | Primer name | F sequence (5’-3’) | R sequence (5’-3’) |
| --- | --- | --- | --- |
| Subcellular localization | *FvWRKY50*-cGFP | ACACCAAATCGACTCTAGTCTAGAATGTTCTTCCCCGGATCATCA | CTCGCCCTTGCTCACCATGGTACCACCTAGCTGGCCTGTGT |
| Y2H | *FvWRKY50*-AD | ATGGCCATGGAGGCCAGTGAATTCATGTTCTTCCCCGGATCATCATCAT | CTACGATTCATCTGCAGCTCGAGCCTAACCTAGCTGGCCTGTGTGATTT |
|  | *FvWRKY50*-BK | CATATGGCCATGGAGGCCGAATTCATGTTCTTCCCCGGATCATCATCAT | TTATGCGGCCGCTGCAGGTCGACGCTAACCTAGCTGGCCTGTGTGATTT |
|  | *FvMAPK3*-BK | CATATGATGAAACTTCAGAGCAGTCACAG | GTCGACTCAAGCATACTCAGGATTGAATG |
|  | *FvMYB10*-AD | CATATGATGGAGGGTTATTTCGGT | GGATCCTCATACGTAGGAGATGTTGACT |
|  | *FvABI4*-BK | CATATGGCCATGGAGGCCGAATTCATGGACGAAGACGACACTTCC | TTATGCGGCCGCTGCAGGTCGACGTCAATCAAATCCTTTAAAATCCAAAAAGAGAGG |
| LUC | nLuc-*FvMAPK3* | GGAGAGAACACGGGGGACGAGCTCGATGGCTGACCACCTCCC | CTTGTAGTCCATTTGTTGGATCCCAGCATACTCAGGATTGAATGCT |
|  | cLuc-*FvWRKY50* | TCGTACGCGTCCCGGGGCGGTACCATGTTCTTCCCCGGATCATCA | CGAACGAAAGCTCTGCAGGTCGACACCTAGCTGGCCTGTGT |
| Protein | *FvWRKY50*-His | ATGGCTGATATCGGATCCGAATTCATGTTCTTCCCCGGATCATCA | GTGGTGGTGGTGGTGGTGCTCGAGACCTAGCTGGCCTGTGT |
| Gene editing target design | *FvWRKY50* CR-T1 | TGATCCGGGGAAGAACATTGTTTTAGAGCTAGAAAT | AATGTTCTTCCCCGGATCATGACCAATGTTGCTCC |
|  | *FvWRKY50* CR-T2 | GCAGCGGTACTGCCAACTAGTTTTAGAGCTAGAAAT | TAGTTGGCAGTACCGCTGCTGACCAATGGTGCTTTG |
| Identification for Gene editing | *Inter spacer*-RT | TCAAACGAGCTTTTACCCTT | GTCTACTATCCAGCGAAACCAC |
|  | *Cas9* | CTGACGCTAACCTCGACAAG | CCGATCTAGTAACATAGATGACACC |
|  | *FvWRKY50* CR 12 | CGACACCTATCTATCTCCCTCTC | CTGGGGAATCTGTTTGCTTTGA |
| qRT-PCR | *Actin* | TGGGTTTGCTGGAGATGAT | CAGTTAGGAGAACTGGGTGC |
|  | *SAUR36* | ACCATACCGTGCCCGATTTC | GTGACTCTGCAACCTAGCCT |
|  | *SAUR50* | TCACCCTGAGTTCCAATGCC | ACGAAAAACGACTTCCTCGC |
|  | *SAUR72* | AGCTTTTGAACAAGTCGGCG | GGAGGTCGCTGATGTCACTG |
|  | *ARF4* | TAAGAGGAGCTGTACAAAGGGC | GGTCATCCCCAACAACCATGA |
|  | *ARF5* | GACCCAAGAGGTTCAGGGTG | GCACACACCCGACAAATTCC |
|  | *MYB44* | ATCCGATGAGGAGCTTTCCG | CAATTCGCTTCACCGCAACA |
|  | *EIN3* | AGGATCAGGGTCCTCCATACA | TGGCACTCTCCTTAGCAGTC |
|  | *JUB1* | GAAGCATTTGCAGGCAAGGA | GGATGGGTCCACAAAGAGCA |
|  | *NAC2* | GCTGGACGATTGGGTTCTGT | CGCACGCCATCAGATTCTTC |
|  | *NAC96* | ACACAAAGCCACAAGATTACCA | ATGGCATTCACCAGATTGGC |
|  | *WRKY6* | TTTCCGGGCTTCAACTCTCC | GACGATCCGCCGATAATGGA |
|  | *WRKY53* | CTCTTAAGCCACCCCGAAGG | TGCCACCAAAGTCTTGCTGA |
|  | *WRKY70* | TGCATGGAGAAAGTACGGACA | TAAGAGAGCCGGTGCAAGTG |
|  | *YUC3* | CCCTGAGGACTTCCCTGAGT | GCAGATGTACTCGACCTCCG |
|  | *YUC4* | CCGGCAAAATAAAGGTGGTGG | CCAACAGCTAGGACAAGGGG |
|  | *YUC6* | AGGGGATCATGGAGTTCGGA | TCCCAGCATCTCTCGTGGTA |
|  | *YUC10* | GAGGTTCTTCACTCGACCCG | CCAAAGCAATCTCCATGCCG |
|  | *FT1* | TGCATGTCATAATTCGCTTG | ATGCATACACACCCACTGGT |
|  | *FT2* | AACACCAGAGAGTTTGCTGAGA | ACGTGGTGTTCCGGGTATAG |
|  | *FT3* | GCCGCCGTCTATTTTAACTG | GCCTTGCAAAATTACTGGTACA |
|  | *TFL1* | CTGGCACCACAGATGCTACA | AACGGCAGCAACAGGAAC |
|  | *SOC1* | ACCCAGATGAGGCGGATAGA | CGCAGAGAATGGAGAGCTCA |
|  | *CO* | CTGTCACCCAAGAACACCGA | ATCCGGGGTCTAGTCTCTGC |
|  | *CHS1* | CATACCCCGACTACTACTTTCGT | CGCACATACTGGGATTCTCTT |
|  | *CHI* | AGCGAAAGCCATTGAAAAGT | CATTTGGTGATTGTGTGAAGAG |
|  | *MYB10* | CAACAGCACCACCACAGACT | GCTTGCCGATTGTACCGTAT |
|  | *C4H* | ACGCTCAACAGAAAGGAGAGAT | TTCGGGGTGGTTCACAA |
|  | *4CL1* | CGTAGACCCTGAAACTGGTG | GTGTAGCCATCCTTCCTTGTC |
|  | *4CL2* | ACGAATCCCTTCCCAGAAA | TCTTTGTCTATGGTCCTCTCAGTC |
|  | *DFR* | ACCCTGAGAACGAAGTGATAAAG | TAAACACCACCCTCCGAACT |
|  | *F3H* | CTTTCGTGGTGAATCTTGGAG | TCGCTATGGACAACCTGCT |
|  | *UFGT* | TAGAGGATGTGTGGAAGATTGGT | CTGTTGTGCGAGTTGTTTTAGTG |
|  | *ANS* | CTTGGCTTGGGATTAGAAGAAG | TGAGGGCATTTTGGGTAGTAGT |
|  | *RAP* | CAAGTTCCAGCAATCGAAGA | TGGGAAGGATCACAAGTTGA |
|  | *RAV1* | AGAGCGCATTCCAAAGTCAG | CACAATCCCCTGTTCCTTCA |
|  | *RIF* | ACGTTCGGAGAGCAAGAGTG | GTTCCGGTCGCCTTCCAATA |
| EMSA | *CHI*-W1610 | CTCATTTTACCAAGTTGACCCCTTCATTTG | CAAATGAAGGGGTCAACTTGGTAAAATGAG |
|  | *DFR*-W290 | CTTATATCCACATCTTGACCGTTCAGTTTATAG | CTATAAACTGAACGGTCAAGATGTGGATATAAG |
|  | *FT3*-W982 | GATCCATTTCCATTGACCAGACTCACCTCAC | GTGAGGTGAGTCTGGTCAATGGAAATGGATC |
|  | *SAUR36*-W1483 | CTTTCCTCTAATCTTGACCCTTCTTTAATC | GATTAAAGAAGGGTCAAGATTAGAGGAAAG |
|  | *SAUR36*-W1636 | CCTTTCCTCCATCCTTGACCTTTAAAACCC | GGGTTTTAAAGGTCAAGGATGGAGGAAAGG |
