# Supplementary figures and images for: FvWRKY50 is an important gene that regulates both vegetative growth and reproductive growth in strawberry
Source: Hortic Res. 2023 May 31;10(7):uhad115. doi: 10.1093/hr/uhad115 (PMC10419500; doi:10.1093/hr/uhad115)

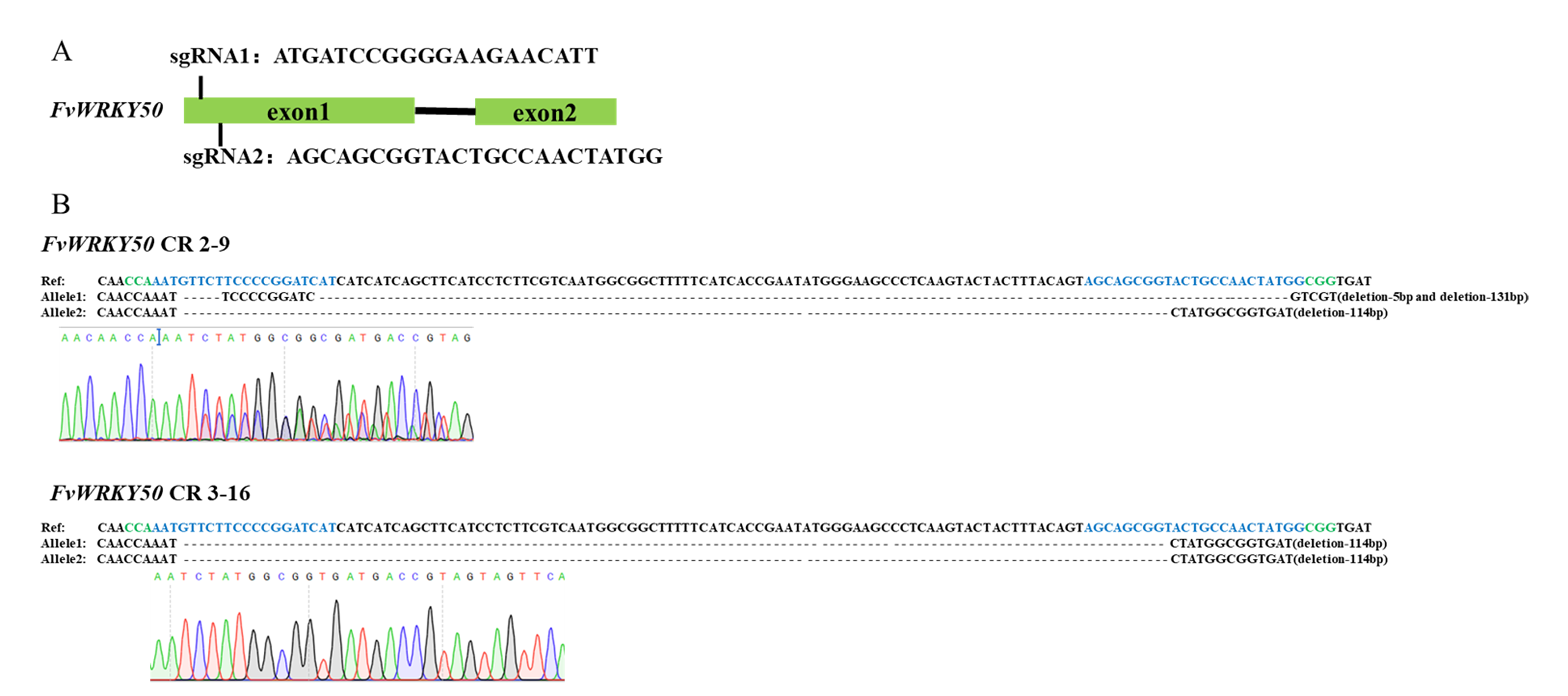

Supplement: Web_Material_uhad115 [file web_material_uhad115.zip › Figure S1.tif]

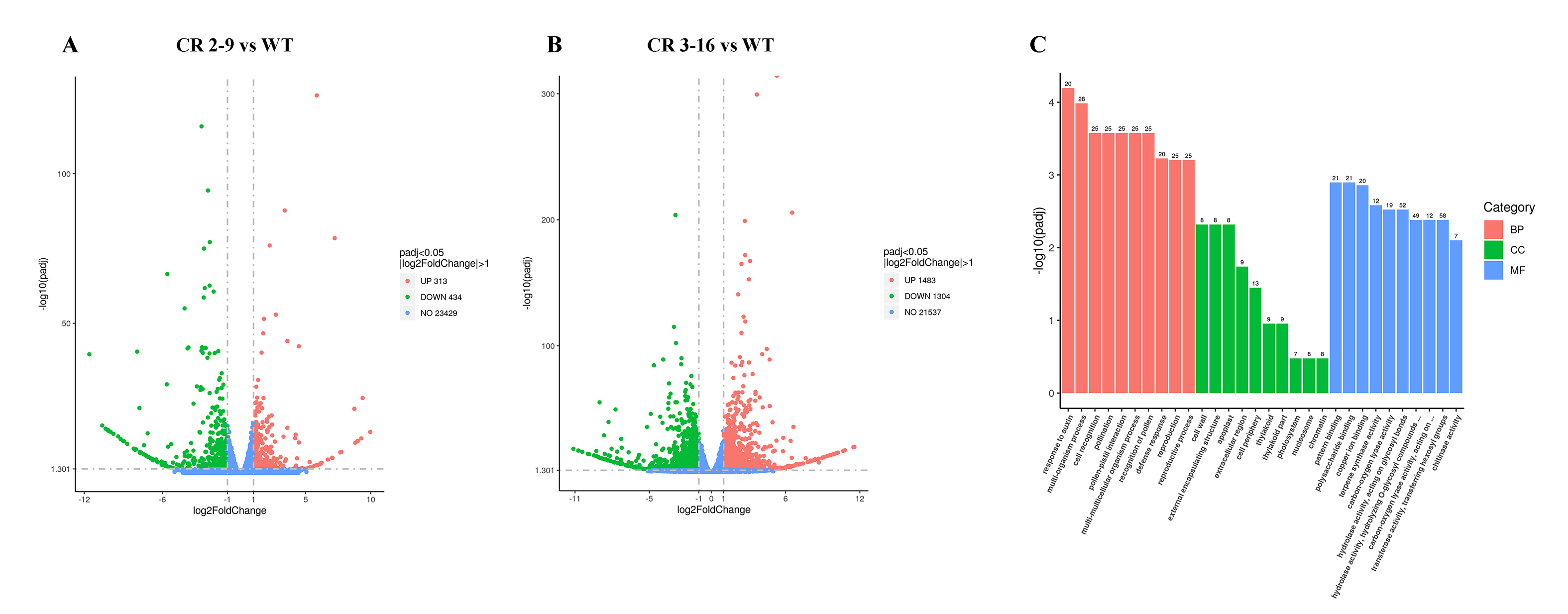

Supplement: Web_Material_uhad115 [file web_material_uhad115.zip › Figure S2.tif]

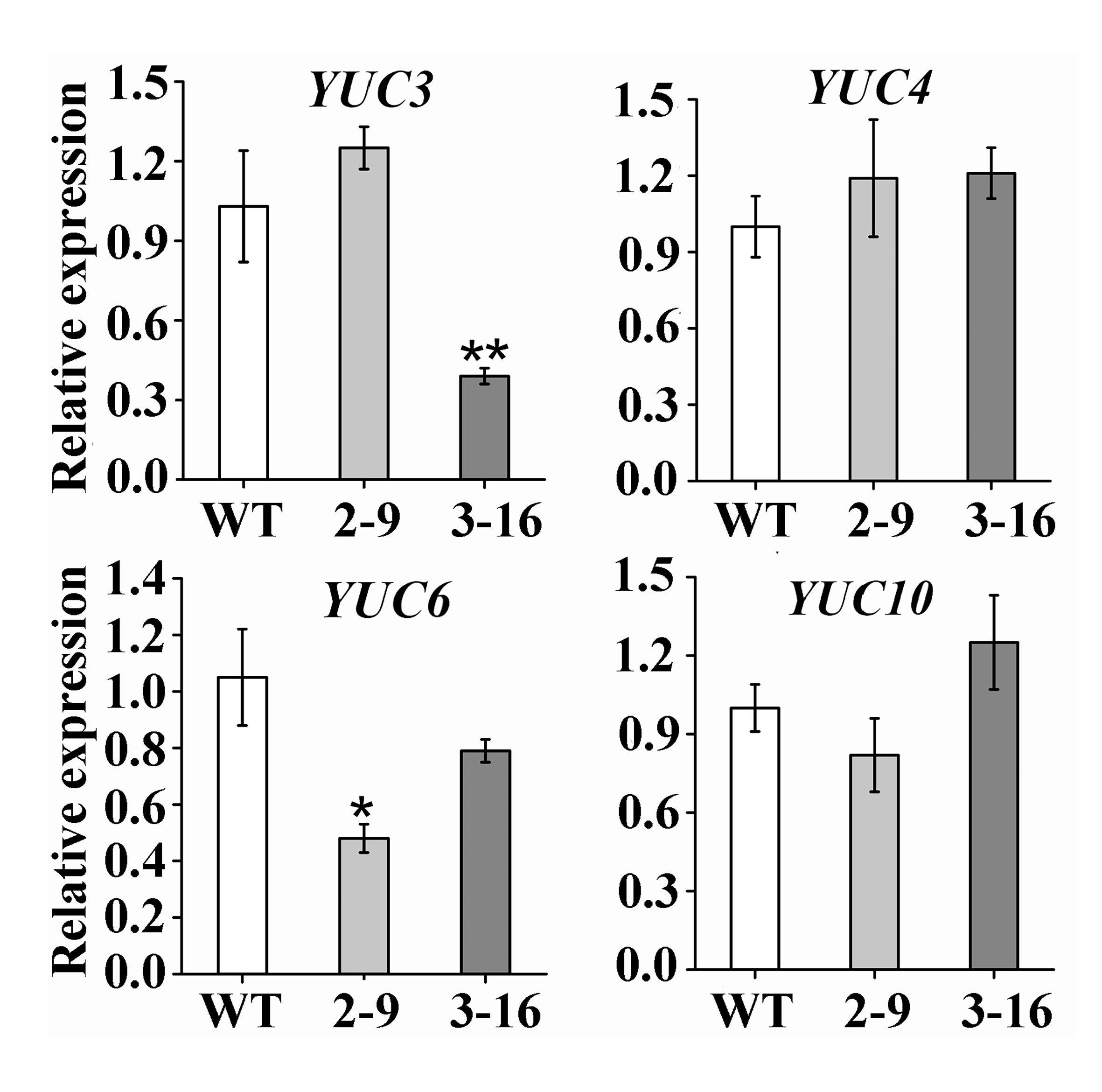

Supplement: Web_Material_uhad115 [file web_material_uhad115.zip › Figure S3.tif]

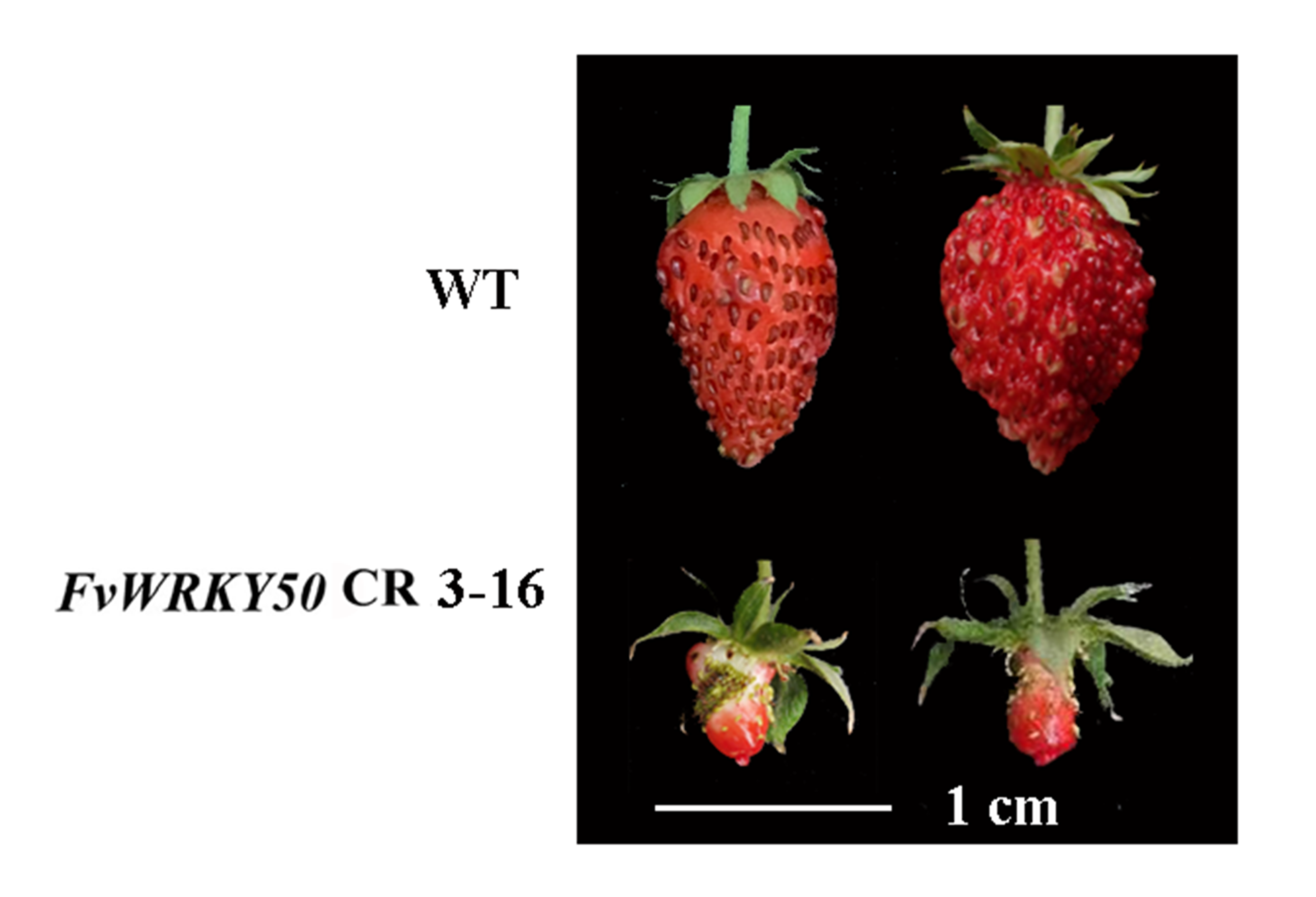

Supplement: Web_Material_uhad115 [file web_material_uhad115.zip › Figure S5.tif]

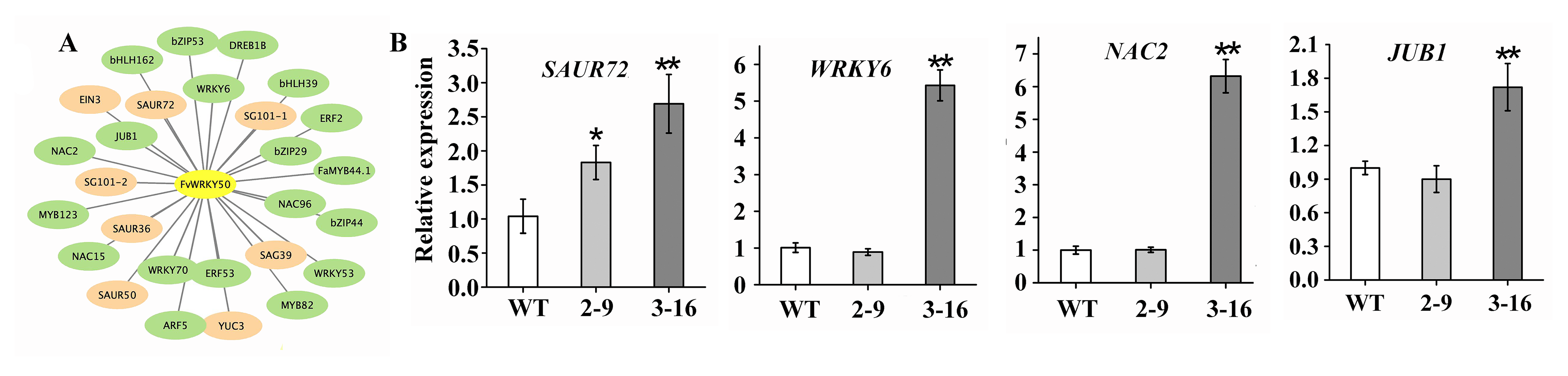

Supplement: Web_Material_uhad115 [file web_material_uhad115.zip › FigureS4.tif]
